# The Obtunding of Dentin

**Published:** 1902-04-15

**Authors:** 


### THE OBTUNDING OF DENTIN.

Among the various chemicals which changes "the constituents of the nerves" by the removal of moisture or by coagulation of the albuminous material, we find solutions of the salts of metals, mineral acids, alkalies, lactic and acetic acid and the compounds of ammonia. With the latter, Dr. Thiesing has experimented. The compounds of ammonia have no specific anesthetic action upon the nerves like cocain, eucain, nirvanin and tropacocain, but they act by withdrawing moisture from the nerves. Therefore, they can not be used for hypodermic injections, as the injection causes severe pain and a paresthesia of a few seconds only. Nevertheless, the anesthesia produced is very efficient. In using these ammonia compounds for local obtundants, the concentration has much to do with the effect. A discoloration of the dentin never results. The inorganic constituents of the tooth never suffer, except in experiment No. 5. The odor of some of the preparations can be partly modified by adding alcoholic solutions of essential oils. A local obtundant is only of value when its action is manifested in about five to ten minutes.

1. *Ammonia bromid:* almost odorless. Application is painless. Ten percent solutions produce after ten minutes only a very weak anesthesia.

2. *Ammonium chlorid* (*sal ammoniac*)*:* applied in twenty percent solution ; after ten minutes only a weak anesthesia.

3. *Ammonium phosphate:* slightly painful, in five to ten minutes perfect anesthesia.

4. *Solution of ammonium acetate:* pains at the very moment of application ; in five minutes total anesthesia.

5. *Solution of ammonium carbonate. Preparation:* sublimation of one part of ammonium chlorid and two parts of chalk and solution in four parts of cold water. The liquor is volatile and has only a faint ammoniacal odor. To be applied in five to twenty percent solutions, acts in five to ten minutes. This preparation can be recommended.

6. *Aqua ammonia:* application is painless in five to twenty percent solutions; in five to ten minutes anesthesia is produced. If applied in thirty to fifty percent solutions it is painful; adding concentrated carbolic acid will almost entirely neutralize this effect.

7. *Alcoholic ammonia:* there is very little difference between this solution and the aqua ammonia.

8. *Anisated aqua ammonia:* the action of this preparation is somewhat less than the alcoholic solution.—Dr. Thiesing, *Deutsche Monatsschrift fuer Zahnheilkunde*

